# Topological supermodes in photonic crystal fiber

**DOI:** 10.1126/sciadv.add3522

**Published:** 2022-12-21

**Authors:** Nathan Roberts, Guido Baardink, Josh Nunn, Peter J. Mosley, Anton Souslov

**Affiliations:** ^1^ Department of Physics, University of Bath, Claverton Down, Bath BA2 7AY, UK.; ^2^ Centre for Photonics and Photonic Materials, University of Bath, Bath BA2 7AY, UK.; ^3^ ORCA Computing Ltd., 30 Eastbourne Terrace, London W2 6LA, UK.

## Abstract

Topological states enable robust transport within disorder-rich media through integer invariants inextricably tied to the transmission of light, sound, or electrons. However, the challenge remains to exploit topological protection in a length-scalable platform such as optical fiber. We demonstrate, through both modeling and experiment, optical fiber that hosts topological supermodes across multiple light-guiding cores. We directly measure the photonic winding number invariant characterizing the bulk and observe topological guidance of visible light over meter length scales. Furthermore, the mechanical flexibility of fiber allows us to reversibly reconfigure the topological state. As the fiber is bent, we find that the edge states first lose their localization and then become relocalized because of disorder. We envision fiber as a scalable platform to explore and exploit topological effects in photonic networks.

## Teaser

Topological photonics is used to design robust modes of light inside structured optical fibre.

## INTRODUCTION

Inspired by electronic quantum materials (*1*), metamaterial structures have recently been used to engineer topological bands for photonic (*2*–*8*) and acoustic (*9*) systems. These bands can be molded to provide backscattering-free propagation (*3*, *5*), disorder-resistant bandgaps (*4*), and protected corner states (*6*). Exploiting these phenomena in photonic chips has enabled topologically robust lasing modes (*10*, *11*) and protected conduits for entangled quantum states (*12*–*16*).

Topological modes are protected because of the topological invariant that characterizes the system. This integer invariant can only change upon closing a bandgap. By directly associating the invariant with physical characteristics such as light propagation, topology can be used to endow a system with protection against fabrication-induced imperfections. The presence of topological modes relies on a few key properties, including the presence of a bandgap and the symmetries of the system. By selecting the necessary ingredients and structure, topological physics can be used to design mode profiles (*12*, *16*), robust pumping (*17*, *18*), and unidirectional transport (*3*, *19*).

For microwaves, topology can be achieved through macroscale structures in combination with resonance effects (*3*, *5*, *6*). However, at optical frequencies, the challenge remains to create waveguides more than a few wavelengths long in which light is both confined by a micrometer-scale structure and topologically protected. For example, arrays of silicon waveguides are limited by scattering loss in the near infrared (*20*), whereas for visible light, state-of-the-art planar fabrication techniques, such as using lasers to inscribe waveguides into a glass chip, are challenging to extend beyond the centimeter scale (*21*). Optical fiber that supports topological modes has previously been proposed (*22*–*26*), but experimental demonstrations have not been seen because of the impossibility of fabricating previous designs with current technology.

Topological fiber could play a crucial role in next-generation quantum networks. Optical fiber is a key enabling technology for long-haul telecommunications networks due to its exceptionally low attenuation (*27*). In the drive to scale up quantum networks, this low attenuation also plays a key role due to the impossibility of noise-free amplification of quantum states (*28*, *29*). In chip-scale experiments, light propagating in topologically protected modes over hundreds of micrometers to millimeters has been shown to inherit robustness from the topological medium (*11*–*15*). Therefore, if the guidance of topological modes could be demonstrated in fiber at length scales of meters to kilometers, this would offer topological robustness within distributed classical and quantum networks. In addition, topological fiber design has the potential to protect the generation of entangled states of light from the detrimental effects of fabrication-induced variations (*30*).

Unlike telecommunications fiber, photonic crystal fiber (PCF) controls the propagation of light using a cross section with wavelength-scale microstructure (*31*, *32*). Tailoring the design of PCF allows unparalleled customization of its dispersion and mode spectrum (*33*). For example, many solid glass cores can be embedded in a periodic cladding of air holes to form a single flexible fiber with a typical diameter of 100 to 250 μm. In this fiber, the individual modes of each core can overlap and collectively form so-called supermodes. This type of multicore structure forms the platform for our topological PCF (TopoPCF) that translates the strategies for topological band engineering into fiber. Fabricating our TopoPCF allows us to observe propagation of edge states with visible light and to use bending for topological mode control. We show that bending the fiber leads to an effective disorder inside our topological state, which we can switch on and off through mechanical reconfiguration. This unique control of topology via disorder exemplifies fiber-based phenomena that are inaccessible in planar waveguide architectures.

**Fig. 1.**
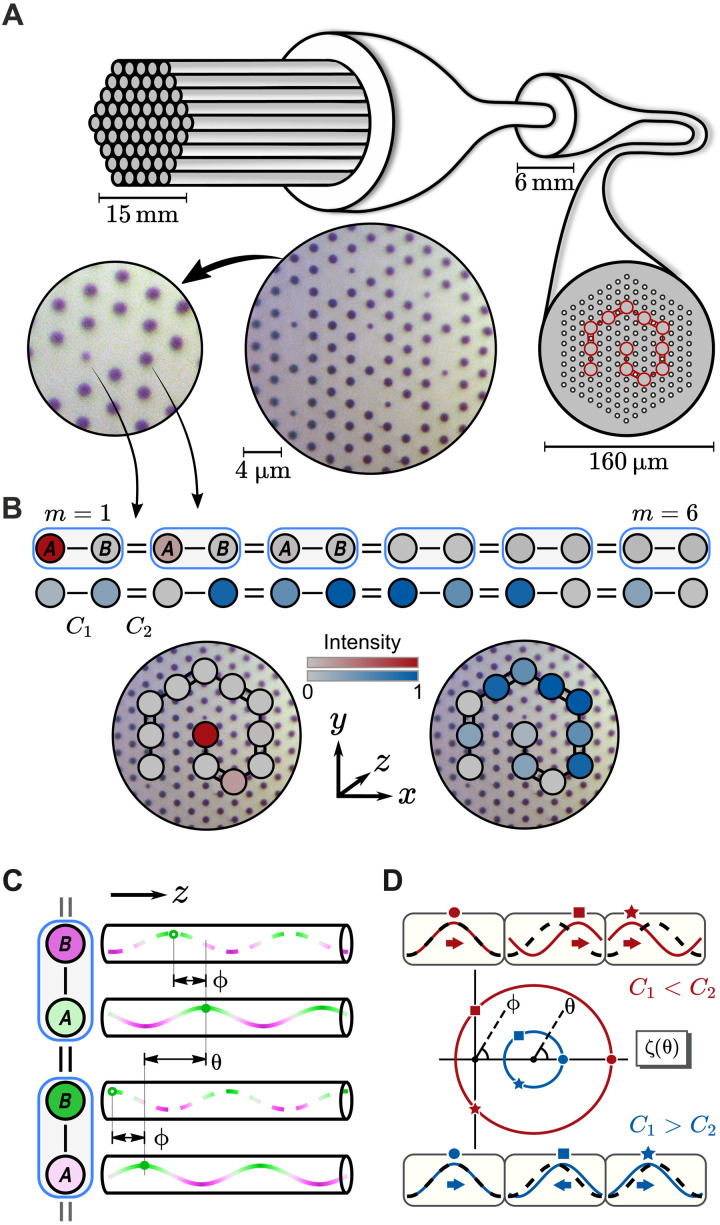
Topological states in fiber. (**A**) Fiber fabrication process: Using a fiber drawing tower, stacked glass capillaries are formed into canes and then fiber. Right: The transverse structure is preserved. Middle: Optical micrograph of fiber cross section with dark air holes and lighter gray glass. Left: Three glass cores and the different size air holes between them. (**B**) Smaller air holes correspond to stronger intercore coupling (double bonds), and larger air holes correspond to weaker coupling (single bonds). A Su-Schrieffer-Heeger (SSH) chain (*34*) has alternating couplings and two cores (*A* and *B*) per unit cell (blue boxes). Its supermodes include topological edge states (top, red) and bulk states (middle, blue). The chains are wrapped into compact spirals within the fiber cross section (bottom). (**C**) The topological invariant ν is encoded in the phase differences (ϕ within a unit cell and θ for neighboring cells) between optical waves propagating in neighboring cores (see main text). (**D**) The function ζ(θ) has argument ϕ and draws a circle in the complex plane. The circle winds around the origin for *C*_1_ < *C*_2_ (topological, red) but not for *C*_1_ > *C*_2_ (trivial, blue). Boxes: Phase difference ϕ between *A* cores (solid) and *B* cores (dashed) for corresponding θ values on the circles.

We create a topological lattice that propagates light over meter scales governed by its micrometer-scale cross section by engineering the coupling between cores (Fig. 1A and the “Fiber fabrication” section). We focus on a canonical model exhibiting a topological invariant, the Su-Schrieffer-Heeger (SSH) chain (*34*), defined by identically shaped cores and alternating intercore coupling strengths. To achieve this, the design alternates between small and large air holes between neighboring cores. The two ends of the chain include an additional small air hole to create a chain of cores uniform in shape. We curl the chain up inside the fiber to form a spiral (Fig. 1, A and B) and observe that both ends of the spiral host topologically protected edge states.

Light is guided by fiber in normal modes. Consider a single optical polarization of the transverse electric field **E** at wavelength λ, which we denote ψ(*x*, *y*) (=*E_x_* or *E_y_*). The eigenvalue equation for ψ(*x*, *y*) and the modal propagation constant β (that is, the longitudinal *z* component of the wave vector) takes a form analogous to the time-independent Schrödinger equation. In this analogy, the square of the local refractive index *n*(*x*, *y*) plays the role of the confining potential for light. Explicitly, the eigensystem reads

$$\nabla_{⊥}^{2}\psi (x,y)+k_{0}^{2}n^{2}\psi (x,y)=\beta^{2}\psi (x,y)$$ (1)

where $\nabla_{⊥}^{2}\equiv \partial_{x}^{2}+\partial_{y}^{2}$ is the transverse Laplacian and *k*_0_ = 2π/λ is the free-space wave number (see derivation in the “Theory of the photonic SSH chain” section). In our system, high symmetry within each core leads to minimal polarization coupling so that the mode profiles are degenerate under rotations of the electromagnetic fields. A solution of Eq. 1 is an electric field profile ψ(*x*, *y*) that propagates unchanged in time *t* along the longitudinal *z* direction as ψ(*x*, *y*)*e*^*i*(ω*t* − β*z*)^, with angular frequency ω = 2π*c*/λ in terms of the speed of light in vacuum, *c*.

The similarity between Eq. 1 and the Schrödinger equation inspires the design of fiber using analogies with electronic band structures. For example, the tight-binding model predicts that for weakly coupled cores, supermodes obey the matrix equation C**u** = Δβ**u**, where Δβ is the change in propagation constant for a supermode relative to the single-core propagation constant and **u** = (*a*_1_, *b*_1_, *a*_2_, *b*_2_, …) is a complex vector describing both amplitudes and phases of the single-core modes constituting the supermode. For the SSH chain (Fig. 1B), the coupling matrix C contains just two (nearest-neighbor) elements: We denote *C*_1_ to be the coupling strength between cores *A_m_* and *B_m_* within the *m*th unit cell and *C*_2_ to be the coupling between *B_m_* and *A*_*m*+1_ across unit cells.

The topological invariant ν is encoded in the phase differences between optical waves propagating in neighboring cores. When the chain is infinitely long, the supermodes **u** can be decomposed into an eigenbasis of supermodes defined by the relations *a*_*m*+1_ = *e*^*i*θ^*a_m_* and *a_m_* = ±*e*^*i*ϕ^*b_m_*. For these normal modes, each core contains the same light intensity, with the phase difference ϕ between the two sublattices within a single unit cell and the phase difference θ between cores on the same sublattice and in neighboring cells, as illustrated in Fig. 1C. In other words, θ is the reciprocal-space variable along the chain. The phase differences ϕ and θ are related via the coupling matrix. In the eigenbasis, C(θ) is a 2 × 2 antidiagonal matrix with entries ζ(θ) = *C*_1_ + *C*_2_*e*^*i*θ^ (and its complex conjugate; see the “Theory of the photonic SSH chain” section). The phase ϕ is fixed as the complex argument of the function ζ(θ), plotted as circles in Fig. 1D. The map ϕ = arg ζ(θ) relates two phases and is, therefore, characterized by an integer invariant called the winding number ν. If the circle ζ(θ) circumscribes the origin, then the winding number ν of the SSH chain is equal to one, which occurs when the intracell coupling is weak, *C*_1_ < *C*_2_. For strong intracell coupling *C*_1_ > *C*_2_, this topological invariant is zero. Mathematically, we compute

$$\nu =\frac{1}{2\pi }\int_{0}^{2\pi }\frac{d\phi }{d\theta }\;d\theta =\{\begin{array}{c} 1\;\;\;if\;C_{1}<C_{2},\\ 0\;\;\;if\;C_{1}>C_{2} \end{array}$$ (2)

Physically, as we change θ by a full period, we note that for ν = 1, the phase difference ϕ between cores *A* and *B* undergoes a full period (2π) shift (Fig. 1D, top boxes). For ν = 0, ϕ stays close to zero (Fig. 1D, bottom boxes).

Topological band theory predicts that an integer invariant characterizing an unbounded system has profound consequences once boundaries are introduced. Because of the alternating coupling coefficients *C*_1_ and *C*_2_ in the SSH chain, a gap opens up (centered at the single-core propagation constant β), which has a gap size of 2∣*C*_1_ − *C*_2_∣ (see the "COMSOL simulations" section). No bulk modes are allowed to have propagation constants inside this bandgap. Nevertheless, the bandgap can host modes localized at a boundary, and the topological invariant encodes information about these boundary modes. A heuristic argument explains this connection. An invariant can only change discontinuously and only when the bandgap closes, such as at a boundary between a topological chain and a trivial cladding. This transition necessitates a closing and reopening of the gap, guaranteeing a mode to exist at the boundary. Because this argument does not rely on the boundary’s geometry, the band topology endows this edge mode with protection against disorder.

## RESULTS AND DISCUSSION

**Fig. 2.**
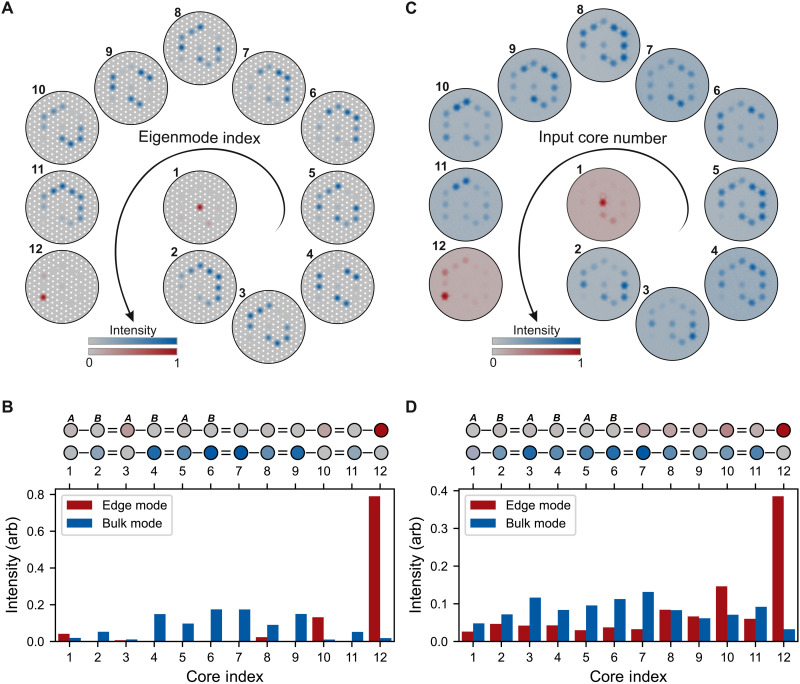
Bulk and edge states. (**A** and **B**) Finite-element simulations. (A) The relative intensity of the electric field for each supermode (doubly degenerate due to polarization). The two edge states (in red) correspond to supermodes that we label 1 and 12. (B) Comparison of the intensity profiles between bulk mode 6 (blue) and edge mode 12 (red). (**C** and **D**) Light propagation experiments: We inject light (wavelength, 636 nm) into a core (input core number) of a TopoPCF with a length of (17.0 ± 0.1) cm and image the output intensity profiles. For input cores 2 to 11, light excites bulk modes and spreads through the chain. For input cores 1 and 12, light remains localized to the topological edge states. (D) Quantitative comparison between the edge mode excited via core 12 and a bulk mode excited via core 4. The topological edge state demonstrates an asymmetry between *A* and *B* sublattices characteristic of the SSH chain.

We fabricated a PCF from silica glass by the stack-and-draw process. Our fiber contains 12 cores coupled to form an SSH chain, as illustrated in Fig. 1A. Using simulations, we estimate the coupling strengths within the fiber to be *C*_1_ = 166m^−1^ and *C*_2_ = 352m^−1^ for light with a wavelength of 700 nm. We verify that our fiber supports chain-edge supermodes through a combination of simulations and experiments shown in Fig. 2. In Fig. 2 (A and B), we numerically solve Maxwell’s full vector equations, finding the propagation constants and field profiles of the supermodes supported by our fiber. These supermodes can be separated into two classes: bulk states (labeled 2 to 11) and exponentially localized edge modes (1 and 12). The bulk states predominately contain contributions from cores that make up the body of the chain and have propagation constants that lie in the bulk bands, above and below a topological bandgap (see the “COMSOL simulations” section). Two topological edge modes are found within this bandgap. These edge modes exist at Δβ ≈ 0, are highly localized to the end of the chain, and are robust to disorder smaller than the bandgap.

In experiments (Fig. 2C), light is injected separately into each of the 12 cores by butt-coupling from a single-mode PCF. For each input condition, we show the intensity distribution after light has propagated through the fiber. When light couples into cores 2 to 11, it excites a combination of bulk modes. When exciting bulk modes, light couples across all cores in the body of the chain and, after propagation, exits as a distribution of intensities across the bulk cores [blue in Fig. 2 (C and D)]. By contrast, light coupled into the two edge cores predominantly excites the topological modes and remains confined to the edge [red in Fig. 2 (C and D)]. Notably, the topological edge states exhibit an asymmetry between the *A* and *B* sublattices, showing an alternating intensity profile characteristic of the SSH chain. This intensity profile arises because of the interaction between the topological edge modes and the constraints enforced by sublattice symmetry.

**Fig. 3.**
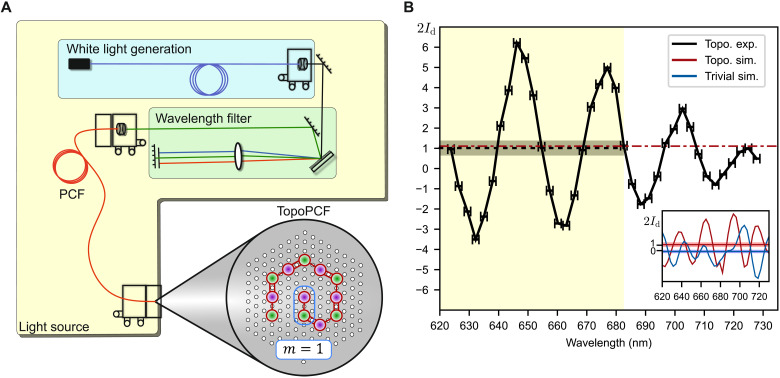
Direct observation of topological invariant. (**A**) Experimental setup consisting of a variable-wavelength light source coupled into a single TopoPCF core (see the “Characterization” section and movies S1 and S2). (**B**) Real-space measurement of winding number. For light injected into a bulk core, the black solid line is twice the weighted intensity difference between the *A* and *B* sublattices, *I*_d_ = ∑*_m_m*(*I_A_* − *I_B_*), where *I*_*A*, *B*_ is the normalized sublattice intensity within one unit cell and the sum is over all unit cells, *m* = 1…6. We vary the wavelength over a 104-nm range and propagate through 17 cm of fiber. We calculate the mean of 2*I*_d_ to be 1.01 ± 0.33 over the highlighted range to confirm the system’s nontrivial winding number (see main text). Error bars are set by wavelength filter resolution, and the gray confidence interval is the SD of the unweighted intensity difference ∑*_m_*(*I_A_* − *I_B_*). Inset: Simulation data for *I*_d_ comparing fiber geometries with winding numbers 0 (blue) and 1 (red). For comparison, the topological simulation average is also plotted as a red dashed line in (B).

To confirm the topological origin of the edge states in Fig. 2, we directly measure the winding number ν (*21*, *35*, *36*). We augment the method from (*21*), with the notable difference that instead of changing the propagation length, we scan over the optical wavelength λ using the setup in Fig. 3A (see movies S1 and S2 for the wavelength-dependent output and the “Characterization” section). With light injected into a core in the bulk, we scan the optical wavelength and observe a change in the intensity distribution at the output of the fiber. This change sees light move from cores belonging to the *A* sublattice to the *B* sublattice. In the “Weighted intensity difference and the winding number” section, we connect this change in sublattice intensity distribution with the winding number that characterizes the system, giving us access to an experimental observable defining the distinct topological invariants. At the output, the topological invariant ν can be computed as twice the weighted intensity difference *I*_d_(λ), also called the mean chiral displacement (*35*), between the *A* and *B* sublattices averaged over all wavelengths λ

$$\nu =2{\left\langle I_{d}(\lambda )\right\rangle }_{\lambda }=2\;{\langle \sum_{m}m(I_{A}-I_{B})\rangle }_{\lambda }$$ (3)

where *I_A_* = ∣*a_m_*∣^2^ and *I_B_* = ∣*b_m_*∣^2^ are the intensities in each sublattice and the sum runs over all unit cells *m* (see derivation in the “Weighted intensity difference and the winding number” section).

We choose a wavelength range sufficiently broad to capture the characteristic oscillations of *I*_d_(λ). Furthermore, we average *I*_d_(λ) over a wavelength subrange such that the quantity ⟨*I*_d_(λ)⟩_λ_ remains invariant under any renumbering of the unit cells. To do so, we pick the wavelength interval over which the measured unweighted intensity difference ∑*_m_*(*I_A_* − *I_B_*) vanishes, as explained in the “Weighted intensity difference and the winding number” section. We then use the SD of this unweighted intensity difference as an unambiguous choice for the error. We plot 2*I*_d_(λ) in Fig. 3B and measure 2〈*I*_d_(λ)〉_λ_ to be 1.01 ± 0.33, confirming bulk topology and bulk-boundary correspondence. The simulations agree with our experimental conclusions and additionally demonstrate that 〈*I*_d_(λ)〉_λ_ = 0 for the topologically trivial case [see Fig. 3B (inset)].

Bulk-boundary correspondence and the localized edge modes are a consequence of the topological invariant characterizing the system. This invariant must always exist, provided the chain displays sublattice symmetry and experiences sufficiently small disorder (that is, smaller than the photonic bandgap). In contrast to a topologically trivial system, the edge modes in our fiber always exist, provided that the invariant remains well defined. Thus, we can think of the edge modes as inheriting the robustness of the topological invariant. This robustness reveals itself when disorder is introduced to the coupling strengths. If the couplings in the chain contain disorder, topologically trivial modes can be perturbed and even destroyed, whereas topological edge states remain immune. For cases that respect sublattice symmetry, the topological mode remains protected as long as the ratio *C*_1_/*C*_2_ is less than unity. By contrast, if the disorder violates sublattice symmetry, then the protection of the edge modes is weaker.

When on-site disorder is introduced into our chain, the sublattice symmetry is broken. Under these conditions, the topological edge mode loses its guaranteed protection and can now be destroyed while the ratio *C*_1_/*C*_2_ is less than unity. However, for the mode to be destroyed, the on-site disorder must be sufficiently large when compared to the size of the bandgap (*37*). In the “Preserving sublattice symmetry” section, we explore the effects of on-site disorder that exist in our fiber. We find that for realistic structural imperfections, the strength of disorder is less than 3.2% of the photonic bandgap. For these small values of the disorder, the edge modes remain identical to the ideal case that respects sublattice symmetry.

**Fig. 4.**
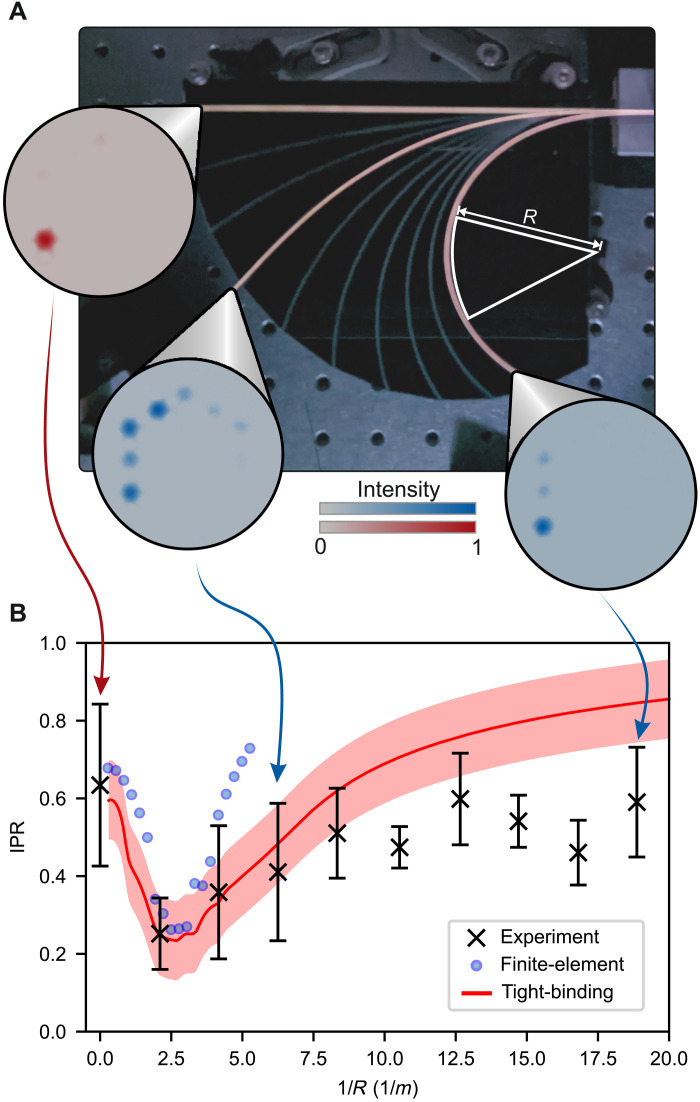
Switchable topology via bending. (**A**) Experimental setup for investigating the effects of bending. In the photo, three fiber segments are lit up to show configurations with decreasing radii of curvature *R*. Insets: Output intensity after light is injected into edge core 12 [arrows indicate values on the curvature axis in (B)]. As curvature increases, topology is lost and the edge mode delocalizes, before trivially relocalizing due to bending-induced disorder. (**B**) IPR [$\mathrm{IPR}=(\sum_{k}I_{k}^{2})/{\left(\sum_{k}I_{k}\right)}^{2}$, where the sum is over all core intensities *I_k_*] quantitatively showing this nonmonotonic behavior (see the “Numerical propagation” section). First, the topological edge state is lost (decreasing IPR) before disorder-induced localization sets in at higher curvature 1/*R* (increasing IPR). Experiment (crosses): Six independent measurements per data point (error bars: SD). Finite-element simulation (circles): A single realization of a bent fiber. Tight-binding model (red line): Mean IPR for eigenmodes in 40,000 realizations of on-site disorder for the SSH chain (shaded interval: SD).

Our fiber platform gives rise to topological phenomena inaccessible in short, rigid waveguides and solid-state devices. One such phenomenon is the reconfigurable control of topological states through geometric bending, only accessible because of the unique physical flexibility of fiber (Fig. 4). In the presence of a bend with radius *R*, the propagation constant of each core changes because of this geometric perturbation by Δβ ≈ 2πΔ*x*/(λ*R*) for cores separated by distance Δ*x* [see the “Numerical propagation” section and (*38*, *39*)]. This geometric control enables us to globally reconfigure the SSH chain with a single macroscopic bend. Such reconfigurability is in contrast to metamaterial systems where individual unit cells can be adapted, but changing the entire lattice presents a challenge.

The changes in propagation constant due to fiber bending map directly onto on-site disorder in the system’s coupling matrix. This mapping enables us to analytically predict the response of the topological edge mode to bending. Because on-site disorder does not preserve the chiral symmetry of the SSH chain, the topological invariant is no longer guaranteed. This loss of symmetry destroys the localizing topological protection and allows light in the edge mode to spread across the bulk modes. We observe this in the insets in Fig. 4A: As the curvature is increased, the topological edge mode becomes delocalized. Upon further bending, this trend begins to reverse and the on-site disorder grows sufficiently large to induce Anderson localization (*40*, *41*), which reconfines light within the initially excited core. To quantify this localization, we calculate the inverse participation ratio (IPR) $\mathrm{IPR}=(\sum_{k}I_{k}^{2})/{\left(\sum_{k}I_{k}\right)}^{2}$, where the sums run over all cores *k* = 1 to 12. We plot the IPR as a function of fiber curvature in Fig. 4. We predict the breakdown of topological protection at the bend radius R^*^, at which the ratio of disorder Δβ to the average coupling strength *C* is near one [Δβ ≈ *C* = (*C*_1_ + *C*_2_)/2]. As found in the “Numerical propagation” section, 1/R^*^ ≈ 1.28λ*C*/(2πΔ*x*) ≈ 3m^−1^, in agreement with Fig. 4B. This bending radius characterizes a simple reversible mechanism for globally breaking and restoring topological protection.

To conclude, we have used the stack-and-draw technique to make hundreds of meters of topological fiber for visible light. We observe the topology and control the corresponding edge states through fiber bending, a mechanism inaccessible to on-chip waveguides. We envision topological fiber as a flexible platform that allows topological effects to be applied in a scalable way to classical and quantum photonic networks.

## MATERIALS AND METHODS

### Theory of the photonic SSH chain

Light propagating in a waveguide is described by Maxwell’s equations in matter with no free charges or currents, constant permeability μ ≈ μ_0_, and spatially varying permittivity ϵ/ϵ_0_ = *n*^2^(*x*, *y*)

$$\nabla \cdot (n^{2}E)=0,\;\nabla \times H=\epsilon_{0}n^{2}\partial_{t}E$$ (4)

together with ∇ · **H** = 0 and ∇ × **E** = −μ_0_*∂_t_***H**. Using the curl-curl identity and the expansion of the first equation in Eq. 4, we obtain the vector wave equation

$$\nabla^{2}E-\frac{n^{2}}{c^{2}}\partial_{t}^{2}E+2\nabla \left(E\cdot \frac{\nabla n}{n}\right)=0$$ (5)

The final term may be ignored in our system, as the variation in the refractive index is negligible everywhere the electric field is non-evanescent (i.e., in the glass). Translation invariance in the *z* direction along the fiber allows us to write solutions to Eq. 5 as superpositions of modes of the form $E=[\psi (x,y)\hat{e}]e^{i(\omega t-\beta z)}$, with angular frequency ω (which is the same in glass as in air), propagation constant β, and a unit vector $\hat{e}$ pointing perpendicular to the fiber. Substituting these modes into Eq. 5 gives the scalar wave equation

$$\nabla_{⊥}^{2}\psi (x,y)+(k_{0}^{2}n^{2}-\beta^{2})\psi (x,y)=0$$ (6)

where $\nabla_{⊥}^{2}\equiv \partial_{x}^{2}+\partial_{y}^{2}$, which is Eq. 1 in the Introduction with *k*_0_ = ω/*c*.

**Fig. 5.**
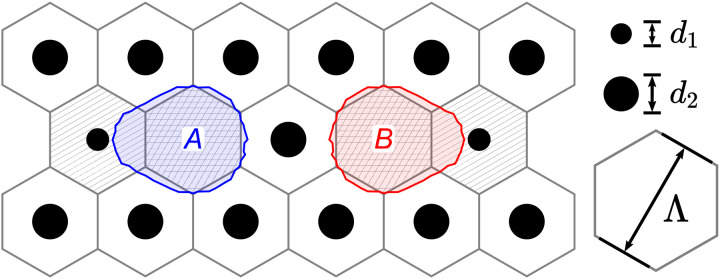
Discretized multicore geometry. The coarse-grained refractive index is *n_g_* on the glass cores (crosshatched) and *n_g_* − *f_j_*δ*n* around the small (linearly hatched) and large (unhatched) air holes, where $f_{j}\propto d_{j}^{2}/\Lambda^{2}$ is the air hole area fraction. A contour line of the single-core mode ψ_2*m* − 1_ localized on an *A* site is drawn in blue. The contour of ψ_2*m*_ on the *B* site is drawn in red. We use exaggerated air hole diameters *d*_1_ = 0.25 μm and *d*_2_ = 0.82 μm and pitch Λ = 4.35 μm.

Let *n_g_* (*n_a_*) be the refractive index of the glass (air) and δ*n* = *n_g_* − *n_a_* their difference. In our multicore fiber, the air hole geometry can be discretized with hexagonal platelets to obtain an effective refractive index *n*(*x*, *y*), which evaluates to *n_g_* on the cores, *n_g_* − *f*_1_δ*n* around the small air hole flanking the core, and *n_g_* − *f*_2_δ*n* around the larger air holes everywhere else. Here, $f_{k}=\frac{\pi }{2\sqrt{3}}d_{k}^{2}/\Lambda^{2}$ is the air hole area fraction in terms of hole diameter *d_k_* and fiber pitch Λ (see Fig. 5).

We expand ψ as a supermode ψ = ∑*_j_u_j_*ψ*_j_* in terms of single-core modes ψ*_j_*. At the first approximation, we can solve for ψ*_j_* on the *j*th core in the absence of other cores. That is, we use Eq. 6 with a coarse-grained refractive index *n_j_*(*x*, *y*) where every *i*th core, with *i* ≠ *j*, is replaced by a large air hole, that is

$$n_{j}(x,y)=n(x,y)-\sum_{i\neq j}\Delta n_{i}(x,y)$$ (7)

where Δ*n_i_* evaluates to *f*_2_δ*n* on the *i*th core and to zero everywhere else. Note that, because the small air hole alternates between being on the left and right sides of the core, the single-core mode on an *A* (*B*) site will be left(right)-heavy, as depicted in Fig. 5.

Writing the shift in propagation constant Δβ for a supermode compared to the single-core β, we subtract the wave equation for the individual cores from the wave equation for the supermode to obtain

$$\sum_{j}u_{j}[2\beta \Delta \beta -k_{0}^{2}(n^{2}-n_{j}^{2})]\psi_{j}=0$$ (8)

Multiplying Eq. 8 on the left by a specific core-mode 〈ψ*_k_*∣ while approximating *n* + *n_j_* ≈ 2*n_g_* and β ≈ *k*_0_*n_g_*, we have

$$\sum_{j}\Delta \beta \langle \psi_{k}|\psi_{j}\rangle u_{j}=\sum_{j}\sum_{i\neq j}k_{0}\langle \psi_{k}|\Delta n_{i}|\psi_{j}\rangle u_{j}$$ (9)

where we use bra-ket notation for convenience. Since the single-core modes are strongly localized, the left-hand side is dominated by 〈ψ*_k_*∣ψ*_k_*〉 ≈ 1, whereas sum on the right-hand side is dominated by 〈ψ_*j*±1_∣Δ*n*_*j*±1_∣ψ*_j_*〉 ≪ 1. Hence, for the dominant terms, we must have Δβ ≪ *k*_0_ and

$$\Delta \beta u_{k}=\sum_{j=k\pm 1}C_{kj}u_{j}$$ (10)

which is our starting point for the tight-binding approach in the main text. These real coupling constants *C_kj_* = *k*_0_〈ψ*_k_*∣Δ*n_k_*∣ψ*_j_*〉 are symmetric because every neighboring pair of single-core modes are reflections of each other. Furthermore, this alternating left/right asymmetry of the single-core modes allows us to define the notation *C*_1_ = *C*_2*m*−1,2*m*_ and *C*_2_ = *C*_2*m*,2*m*+1_ with *C*_1_ < *C*_2_.

We rewrite Eq. 10 in bra-ket notation as C∣**u**〉 = Δβ∣**u**〉 and the ket ∣**u**〉 = ∑*_m_a_m_*∣*A_m_*〉 + *b_m_*∣*B_m_*〉 in terms of *a_m_* = *u*_2*m*−1_ and *b_m_* = *u*_2*m*_. Then, our coupling matrix can be expressed as

$$C=\sum_{m=1}^{M}C_{1}|B_{m}\rangle \langle A_{m}|C_{2}|A_{m+1}\rangle \langle B_{m}|+h.c.$$ (11)

Note that the second term introduces coupling between different unit cells (different *m*). To simplify the analysis, we use a discrete Fourier transform (DFT) to switch to a different basis enumerated by discrete angles θ*_k_* = 2π*k*/*M* for *k* = 1, …, *M*

$$|A(\theta_{k}),B(\theta_{k})\rangle =\frac{1}{\sqrt{M}}\sum_{m=1}^{M}e^{im\theta_{k}}|A_{m},B_{m}\rangle$$ (12)

In this basis, C couples only between states with the same θ*_k_*

$$C=\sum_{k=1}^{M}\zeta (\theta_{k})|B(\theta_{k})\rangle \langle A(\theta_{k})|+h.c.$$ (13)

where ζ(θ*_k_*) = *C*_1_ + *C*_2_*e*^*i*θ*_k_*^. Note that Eq. 11 contains coupling to the extraneous core *A*_*M*+1_, which the DFT equates with core *A*_1_. To remove these periodic boundary conditions, the standard method is to state that the boundary is sufficiently far away by sending *M* → ∞.

In the continuum limit, θ lives on the interval [0,2π]. We use Eq. 13 to solve the eigenproblem C∣**u**〉 = Δβ∣**u**〉 and obtain two solutions for every θ, given by eigenvectors

$$|u_{\pm }(\theta )\rangle \propto \pm e^{i\phi (\theta )}|A(\theta )\rangle +|B(\theta )\rangle$$ (14)

where ϕ(θ) = arg ζ(θ), and eigenvalues

$$\Delta \beta_{\pm }(\theta )=\pm |\zeta (\theta )|$$ (15)

The two bands of eigenvalues are separated by a gap at θ = π of size 2∣*C*_1_ − *C*_2_∣ around the single-core propagation constant β. Inside this gap, no supermodes can propagate through the bulk of the chain. In terms of the components of **u** = (*a*_1_, *b*_1_, *a*_2_, *b*_2_, …), the eigenvectors read

$$\left(\begin{array}{c} a_{m}\\ b_{m} \end{array}\right)=\frac{1}{\sqrt{2M}}\left(\begin{array}{c} \pm e^{i\phi (\theta )}\\ 1 \end{array}\right)e^{im\theta }$$ (16)

which forms the starting point of our definition of topological invariant ν in the Introduction, as defined in Eq. 2 and illustrated in Fig. 1 (C and D).

### COMSOL simulations

We numerically solve the full Maxwell’s vector equations defined in the “Theory of the photonic SSH chain” section to find modes of the form $E(x,y,z)=\tilde{E}(x,y)e^{i(\omega t-\beta z)}$, including the propagation constants β and transverse field profiles $\tilde{E}(x,y)$ of the normal modes supported by the fiber cross section. We confirm that the coupling to the polarization of light is sufficiently weak for modes to take the form $E=[\psi (x,y)\hat{e}]e^{i(\omega t-\beta z)}$ for arbitrary in-plane polarization $\hat{e}$, where we take the time variable *t* to be fixed. We solve this equation for a finite cross section selected to match the parameters of the fabricated fiber. Using COMSOL Multiphysics, we run finite-element analysis simulations with varying meshes, materials, and boundary conditions. The COMSOL design process is illustrated in fig. S1.

In all our simulations, a bipartite mesh was used, with the core sections featuring a denser mesh than the surrounding cladding. The boundary conditions remain fixed for all simulations: A perfect electric conductor boundary was defined at the perimeter of the cladding. We model the refractive index of silica by implementing in COMSOL a wavelength-dependent Sellmeier equation with the refractive index of the air holes taking a constant value of 1.

**Fig. 6.**
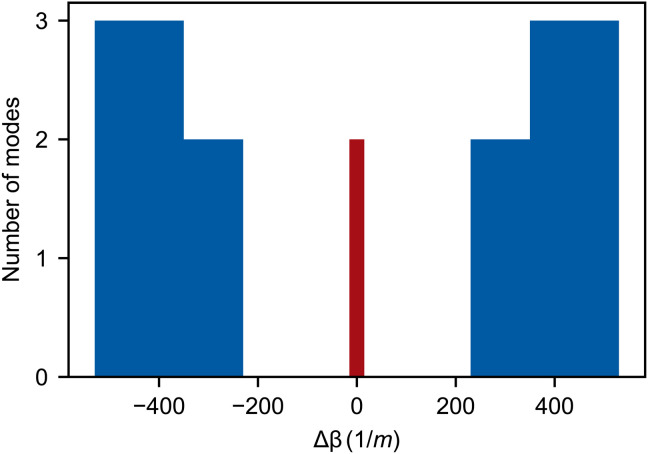
Propagation constant distribution for simulated supermodes. A histogram showing the distribution of the propagation constants (for a single polarization) computed in simulations and plotted in Fig. 2A. The histogram highlights the gap between bulk bands (blue) in the middle of which we find the two topological edge modes (red). As predicted by the analytic theory, these modes exist at Δβ ≈ 0.

After specifying materials, mesh sizes, and boundaries, the spectrum of normal modes is calculated. For our TopoPCF, these modes are plotted in Fig. 6. The electric field data from these modes are plotted as ∣*E*(*x*, *y*)∣^2^/max(∣*E*∣^2^) to show relative field intensities and compare to experimental observations. In Fig. 2A, we directly plot the simulated modes, and in Figs. 2B, 3B, and 4B, we integrate the intensity across each light-guiding core. We use this integration to normalize the supermode vector **u** such that the intensities sum to one.

**Fig. 7.**
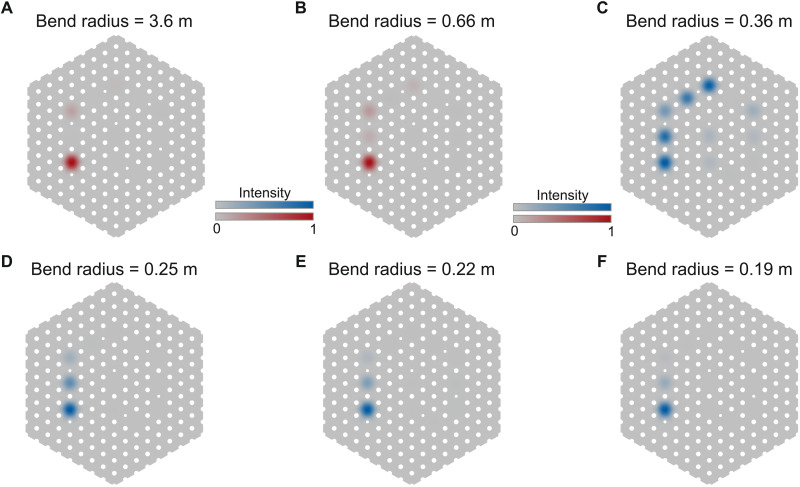
Bending simulations. Results from finite-element simulations of eigenmodes for different bending radii. (**A**) Topological edge mode at large bend radius. (**B**) As we decrease the bend radius, the topological edge mode becomes less localized. (**C**) When the on-site disorder induced by the bend becomes comparable to the coupling strength, the topological protection is broken and the mode becomes delocalized. (**D**) As the bend radius decreases further, the mode begins to relocalize because of Anderson localization. (**E** and **F**) The mode continues to localize further as the bend radius is decreased.

To simulate fiber bending, the spatial profile *n*(*x*, *y*) of the refractive index of the material was modified, which allows normal modes to be calculated in a bent fiber using our two-dimensional (2D) model of the cross section. Following the work in (*38*, *39*), the refractive index was modified to be *n*_bend_ = *n*_silica_(1 + *q*/*R*_eff_), where *R*_eff_ = 1.28*R*_bend_ and *q* is the perpendicular distance to the bend axis, to accommodate the change that light would experience in a bent fiber. As highlighted in (*38*), an effective bending radius *R*_eff_ was used to account for both geometric and stress-optic effects. A selection of the intensity plots found through bending simulations can be found in Fig. 7.

**Fig. 8.**
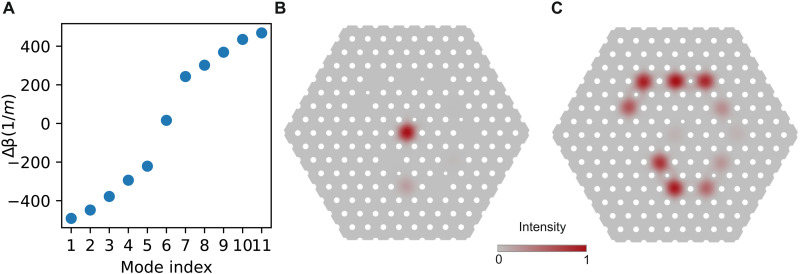
The 11-core SSH chain. (**A**) Propagation constants for the 11 supermodes of the shortened chain. The plot highlights the presence of a bandgap featuring a single zero mode, localized to just one edge of the chain. (**B**) Intensity plot of the topological edge mode lying in the bandgap. This edge mode stays fixed to the core at the center of the fiber because, at that edge of the chain, the topological unit cell is not cut. That is, the boundary conditions at the center are the same as in the 12-core fiber that we fabricated, but the boundary conditions at the other end of the chain are different. (**C**) Intensity plot for a bulk mode, which now contains contributions from core 11 at the outer end of the chain. As expected, because the unit cell is cut at this end, this edge no longer hosts a topological state.

The edges of our SSH chain define the system’s boundary conditions by determining whether the end of the chain cuts a topological unit cell. For systems with an even number of cores, symmetry necessitates that both ends feature the same boundary conditions. However, if the chain features an odd number of cores, then one side will cut a topological unit cell and subsequently lose its edge mode. Once the system features an odd number of cores, there is no way to remove the topological edge (while respecting sublattice symmetry). The topological edge state can simply be moved from one end to the other by adjusting the coupling strengths and, thus, unit cell definitions. In Fig. 8, we show through numerical simulations the difference under boundary conditions and the presence of a single edge mode. Figure 8A shows the propagation constants of an 11-core chain that both starts and ends on a core belonging to the B sublattice. The key feature in Fig. 8A is the single mode lying in the bandgap. We plot the intensity distribution of this mode in Fig. 8B to show that only the topological edge mode at the center of the fiber now remains. Figure 8C shows that core 11 (the outer end core of the chain) now contributes to bulk modes of the system and has been shifted out of the bandgap. While this change destroys a single topological edge mode, sublattice symmetry changes are needed to destroy the topology everywhere in the chain.

### Preserving sublattice symmetry

To confirm that our results are not due to localized disorder, we must ensure that all cores are sufficiently similar. Local structural uniformity ensures that all of the light-guiding cores have the same propagation constant. Uniform propagation constants are required to make a direct mapping to the tight-binding model. In our system, all cores have the same immediate environment (up to rotational symmetry). However, because of imperfections induced in fabrication and the perturbations that each core may experience from other cores in the fiber, this symmetry cannot be guaranteed in practice. That is, chiral and translational symmetries are only approximate in our fiber. The lack of hard symmetry constraints means that, theoretically, a topological phase transition can occur without the full closing of the bandgap. However, the topological invariant that characterizes the system remains well defined as long as the bandgap is much larger than the variation in on-site potentials (*37*).

**Fig. 9.**
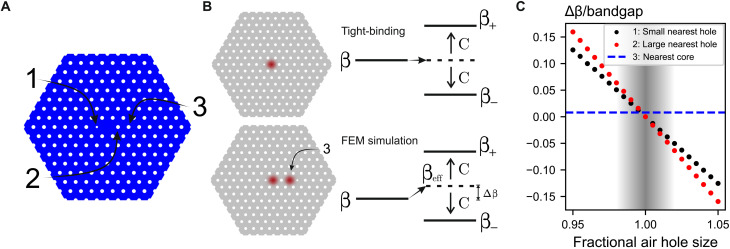
On-site potential changes due to core fluctuations. (**A**) Cross section of a single-core simulation used to investigate the effects of fabrication defects. Labels 1, 2, and 3 correspond to the different core-changing perturbations that we consider: Label 1 corresponds to the change in radius of the small air hole (mean diameter, 1.0 μm); label 2 corresponds to the change in radius of the large air hole (mean diameter, 1.6 μm), and label 3 corresponds to the introduction of a neighboring core. (**B**) A schematic representation of how the presence of a neighboring core induces the on-site change Δβ in the propagation constant. This change can be obtained by first calculating the average propagation constant of two supermodes in both the finite element method (FEM) COMSOL simulations and the tight-binding model. The change Δβ is then estimated by taking the difference between these two averages. (**C**) We compare the on-site change induced by the three different types of perturbation (labeled 1, 2, and 3). The gray region highlights realistic bounds on fabrication disorder, which allows us to estimate the width of the distribution for Δβ/(bandgap size) for a realistic fiber, which we find to have a small width of 3.2%.

We confirm by numerical simulation that on-site disorder is two orders of magnitude smaller than the bandgap size when considering experimental parameters. We consider three changes in geometry that influence the propagation constant of a single core, shown in Fig. 9. The first two modifications could arise as a result of fabrication imperfections. The two different sizes of air holes immediately adjacent to a core could be over- or underinflated. This would directly change the propagation constant and, hence, the effective on-site potential. We model in COMSOL the effects of changes in air hole diameter of up to 5%; however, we typically observe less than 1% change in the geometric parameters of fabricated fiber, as demonstrated in (*30*). We find that with a 1% change to air hole radius, the core’s propagation constant changes by only 3.2% of the bandgap size.

In Fig. 9B, we consider how the propagation constant of a core changes when introducing a new neighboring core. When a second core is coupled to the first, the band structure splits into two supermodes. In the tight-binding approximation, the cores do not affect each other’s on-site potentials and the propagation constants of the supermodes are equally split above and below the single core’s propagation constant (see Fig. 9B). To estimate the shift in the single-core propagation constant from our COMSOL simulations, we then take the average of the two supermodes. This shift between the averaged supermode propagation constant and the single-core case allows us to estimate the change in on-site potential as a result of the second core. We calculate this shift of the on-site potential to be 0.8% of the system’s bandgap size.

**Fig. 10.**
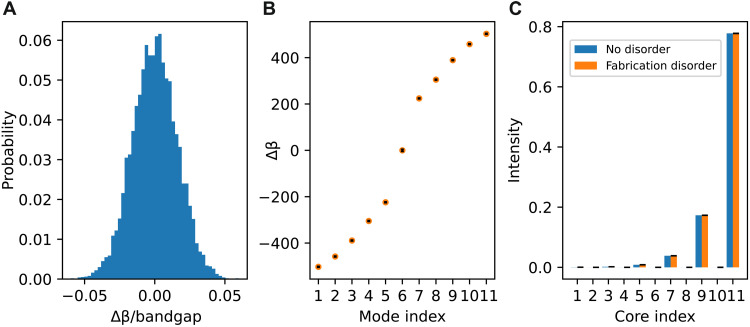
Effects of on-site potential changes. (**A**) Using the width estimate defined in Fig. 9C, we include a normal distribution of on-site disorder in our tight-binding model. This graph shows the distribution of on-site disorder for the 10,000 instances for which data are shown in this figure. (**B**) Average propagation constant in the presence of disorder compared to the system with no disorder. The black lines show the SD (smaller than symbol size). We conclude that realistic experimental disorder does not change any of the conclusions of our work. (**C**) Intensity profile for a single edge mode, averaged over the disorder and compared to the case without disorder. The black lines show SD (which is so small compared to the average intensity that it is not visible).

To verify the assumption that the invariant remains protected, we now insert these small changes into the tight-binding model and investigate the resultant mode structure. In Fig. 10A, we define the distribution from which our random disorder is drawn. This distribution features an SD of 1.6% change in propagation constant when compared to the bandgap. Figure 10B shows the average propagation constants for the modes of our disordered system, with the black lines showing the SD. We take 10,000 iterations of random on-site disorder from our distribution in Fig. 10A and apply these to our tight-binding model. Figure 10C shows the average edge mode intensity distribution found over the same 10,000 iterations, with the black lines again showing the SD. Figure 10 (B and C) shows no significant deviations from the ideal case without disorder and still demonstrates a complete topological bandgap and a sublattice-polarized edge mode. In Fig. 10 (B and C), we use an 11-core SSH chain in place of our 12-core experimental system. This calculation was chosen because an 11-core chain only has one topological edge mode, at Δβ = 0.

### Numerical propagation

Investigating light propagation begins with defining an input vector **u**(*z* = 0), describing the complex amplitude of each individual core mode in the initial excitation. The overlap integral between this input vector and each of the 12 supermodes (**u**_1_, **u**_2_, …, **u**_12_) gives the contribution of each supermode present in the initial excitation. By definition, these supermodes propagate along the fiber with only a change in phase (at any fixed time *t*): **u**_1_(*z*) = **u**_1_(0)*e*^−*i*β_1*z*_^. By multiplying each supermode by its contribution and phase change, we find the final description of the supermode at a distance *z* from the input location. After finding the final vectors describing each supermode at the desired distance, we sum these contributions to find the total field distribution in the supermode basis. To return to the core basis, we then perform a second set of overlap integrals for this final supermode vector with each individual core vector, returning a vector containing the contribution of each core to the final state. Multiplying this final state by its complex conjugate returns a vector **I**(*z*) = (∣*a*_1_∣^2^, ∣*b*_1_∣^2^, ∣*a*_2_∣^2^, ∣*b*_2_∣^2^, …) of relative intensities, describing how much light is in each core in the chain at distance *z*.

In addition to plotting the propagation of light, the benefit of this numerical propagation is that it allows simulation of the wavelength dependence of the sublattice intensity difference *I*_d_(λ) used in Eq. 3. By finding the supermodes for wavelengths in the range shown in Fig. 3B, numerical propagation can be used to model the output of the fiber after propagating through a given distance *z*. This semi-analytical method allowed us to make use of the detailed simulation results without computationally expensive 3D models, while giving more accurate predictions than a purely analytical approach based on the tight-binding model.

Following the “COMSOL simulations” section, the change in propagation constant is

$$\Delta \beta =2\pi \Delta x/(R_{eff}\lambda )=2\pi \Delta x/(1.28R_{bend}\lambda )$$ (17)

We modify the coupling matrix C using diagonal elements corresponding to Δβ for each core. In general, each core’s Δβ will have a different value, which, in addition, depends on the bending axis, therefore playing the role of (diagonal) on-site disorder. To separate the general effect of bending from the specific geometry of our design, we now bend this fiber over a range of different bending directions and average the results.

We consider 40,000 different bending directions for each point on the *x* axis and find the eigenvectors for every system. The IPR values for these eigenvectors are plotted as a 2D histogram in fig. S2. Here, we see that at low curvature, there are two populations of modes with different IPRs: the localized topological edge states with IPR ≈0.6 and the bulk states with IPR ≈0.15. As we are interested in the breakdown of topologically protected states, we now discard the bulk states and focus on the effects displayed by the edge states. We select the two eigenmodes corresponding to topological edge modes (in every bend realization) and average their IPRs over all 40,000 points at each step, leaving us with the average effect of a bend on the IPR of a topological edge mode. Averaging the variances of each set of 40,000 bends and then taking the square root allow us to fit an average SD for the entire distribution, as shown in the curve labeled “tight-binding” in Fig. 3B.

To estimate the point at which the topological edge mode delocalizes, we consider the case when the on-site disorder becomes equal to the average coupling strength (Δβ ≈ *C* = (*C*_1_ + *C*_2_)/2). At wavelength λ = 500 nm, the critical curvature corresponding to this disorder is found to be: $1/R_{bend}^{*}\approx 1.28\lambda C/(2\pi \Delta x)\approx 3m^{-1}$, which is the value quoted in the "Results and discussion" section.

### Fiber fabrication

The TopoPCF shown in Fig. 1A was fabricated using the stack-and-draw process. The first step was to create a macroscopic preform consisting of silica rods and tubes, stacked by hand in the geometry corresponding to the fiber design. As the design required two air hole sizes, we used silica tubes with two different ratios of outer-to-inner diameter. The precursor to the larger air holes in the cladding was a tube with outer/inner diameter of 25 mm/10 mm, whereas the smaller air holes, found in between every other pair of cores, were created from a 10-mm/3-mm tube. The cores were formed from a solid silica rod. Each element was drawn to a diameter of 1.05 mm and stacked into a regular triangular array with the desired layout.

The completed stack was inserted into a 25-mm/17-mm silica tube and drawn into 4-mm-diameter canes, containing the hole pattern required for the fiber. Fiber preforms were created by jacketing canes in 6-mm/4-mm tubes with brass fittings to enable pressure to be applied to the holes in the cane to prevent collapse during the fiber draw.

The fiber was drawn to a diameter of 162 μm with a high-index ultraviolet-cured polymer coating applied during the draw to prevent damage. The PCF used in this work had a pitch (the center-to-center distance for two neighboring air holes) of approximately 4.4 μm, with a large air hole diameter of approximately 1.6 μm and small air hole diameter of approximately 1.0 μm.

### Characterization

Figure 3A highlights the key features of our experimental setup. We used a 1064-nm subnanosecond microchip laser to generate a broadband supercontinuum spanning the visible and near infrared in a length of single-core PCF. This provided white light in a single spatial mode from which a tunable wavelength range was selected by the monochromator shown in the green box in Fig. 3A. The monochromator was formed of a folded 4-f line containing a reflective diffraction grating and a mechanically adjustable slit to pass the desired wavelength range with a bandwidth of approximately 2 nm in the spectral region of interest. The pass-band was collected by a length of endlessly single-mode (ESM) PCF for delivery to the TopoPCF. The pitch of the ESM PCF was approximately 2 μm, which is less than half that of the TopoPCF, enabling the ESM PCF to selectively address individual cores in the TopoPCF by butt-coupling. After propagation, a magnified near-field image of the output of the TopoPCF was formed on the camera sensor by an aspheric lens. The ESM PCF was positioned with a three-axis flexure translation stage to couple light into each core of the TopoPCF sequentially to obtain output intensity distributions for all bulk and edge excitations shown in Fig. 2C. The total intensity at the output of each core was calculated from the images using Python.

To make the direct observation of the topological invariant shown in Fig. 3B, we first established a wavelength range over which the bandwidth of the input light remained approximately constant. We tuned the pass-band of the monochromator over a 104-nm range between 632.8 and 727.8 nm, and using an optical spectrum analyzer, we observed that the bandwidth of the transmitted light did not change significantly. This process also enabled the slit position of the monochromator to be calibrated with transmission wavelength. After calibration, the light was coupled from the ESM PCF into core 2 in the TopoPCF. Small wavelength steps (average step size, 2.7 nm) were made by varying the slit position in the monochromator without changing any other components of the system. At each wavelength step, the output near-field image from the TopoPCF was recorded. From these images, the total intensity difference in each unit cell was found.

The bend measurements required additional components in the optical setup. An acrylic board was laser-etched to create circular grooves with 10 different radii, corresponding to the curvatures seen in Fig. 4B. The board was then fixed in place between the input fiber and the camera to hold the TopoPCF at specific bends. After coupling light into core 12, the input was held constant while the fiber was moved between grooves in the board. For each curvature, the output end of the TopoPCF was imaged onto the camera. This process was repeated three times for two different rotational orientations of fiber to minimize any specific geometric effects from the axis of the bend. The IPR was calculated from the total intensity *I_k_* in each core, where *k* = 1…12, using $\mathrm{IPR}=(\sum_{k}I_{k}^{2})/{\left(\sum_{k}I_{k}\right)}^{2}$. The results are plotted in Fig. 4B.

### Weighted intensity difference and the winding number

Here, we derive Eq. 3, which allows us to compute the winding number from propagation measurements. We then justify our analysis for this experimental winding number computation, including the error estimate.

We consider how the intensity distribution changes as light propagates through the system, from being input into a single *A* or *B* core in a unit cell labeled by the integer *m*^⋆^. We define two operators for the weighted intensity difference I_d_ and the unweighted intensity difference ${\tilde{I}}_{d}$ as

$$I_{d}=\sum_{m}m(I_{\mathrm{Am}}-I_{\mathrm{Bm}})\;\mathrm{and}\;\;{\tilde{I}}_{d}=\sum_{m}(I_{\mathrm{Am}}-I_{\mathrm{Bm}})$$ (18)

where *I_Am_* = ∣*A_m_*〉〈*A_m_*∣ is the intensity of light in the *A* site (and *I_Bm_* = ∣*B_m_*〉〈*B_m_*∣ is the intensity in the *B* site) of the *m*th unit cell and *m* runs from 1 to 6 in our fiber. The evolution of these quantities along the fiber can be described in terms of the coupling matrix C multiplied by the propagation distance *z* to yield the expectation value *I*_d_(*z*)

$$I_{d}(z)=\langle u(z)|I_{d}|u(z)\rangle ,\;\mathrm{where}|u(z)\rangle =e^{-iCz}|u(0)\rangle$$ (19)

We take a Fourier transform along the chain as if the chain were infinitely long, as described in the “Theory of the photonic SSH chain” section. Identifying 〈*A*(θ)∣ = (1,0) and 〈*B*(θ)∣ = (0,1) in Fourier space, we replace *m* → *i∂*_θ_ and I*_Am_* − I*_Bm_* → σ*_z_*. We write the vector **ζ** = (Reζ, Imζ, 0) using complex notation, such that C = **ζ·σ**. Here, **σ** = (σ*_x_*, σ*_y_*, σ*_z_*) is the vector of Pauli matrices

$$\sigma_{x}=\left(\begin{array}{c} 0\;1\\ 1\;0 \end{array}\right),\;\sigma_{y}=\left(\begin{array}{c} 0\;i\\ -i\;0 \end{array}\right),\;\sigma_{z}=\left(\begin{array}{cc} 1 & 0\\ 0 & -1 \end{array}\right)$$ (20)

We note that our definition of *I*_d_(*z*) depends on the labeling of the input cell *m*^⋆^. Supposing, for notational simplicity, that the light starts out in the *B* site of this unit cell, i.e. ∣**u**(0)〉 = ∣*B*_*m*^⋆^_〉, we have

$$\begin{array}{r} I_{d}(z)=\frac{1}{2\pi }\int_{0}^{2\pi }(0\;1)(e^{{+im}^{⋆}\theta }e^{+i(\zeta \cdot \sigma )z}\;i\partial_{\theta }\sigma_{z}\;e^{-i(\zeta \cdot \sigma )z}e^{-{im}^{⋆}\theta })\left(\begin{array}{c} 0\\ 1 \end{array}\right)\;d\theta \end{array}$$ (21)

$$\begin{array}{r} =\frac{1}{2\pi }\int_{0}^{2\pi }(0\;1)(e^{+i(\zeta \cdot \sigma )z}\;(i\partial_{\theta }+m^{⋆})\sigma_{z}e^{-i(\zeta \cdot \sigma )z})\left(\begin{array}{c} 0\\ 1 \end{array}\right)\;d\theta \end{array}$$ (22)

$$=I_{d}^{⋆}(z)+m^{⋆}{\tilde{I}}_{d}(z)$$ (23)

Here, $I_{d}^{⋆}=\sum_{m}(m-m^{⋆})(I_{Am}-I_{Bm})$ is the centralized weighted intensity difference. Note that $I_{d}=I_{d}^{⋆}$ if the input cell is labeled *m*^⋆^ = 0.

We first show that the winding number ν can be computed using the wavelength average of $I_{d}^{⋆}(z)$. To start, the integrand of $I_{d}^{⋆}(z)$ can be simplified by first expanding the exponent of the Pauli vector

$$e^{\pm i(\zeta \cdot \sigma )z}=\cos\;(|\zeta |z)1\pm i\sin\;(|\zeta |z)(\hat{n}\cdot \sigma )$$ (24)

where $\hat{n}=\zeta /∥\zeta ∥=(\cos\phi ,\sin\phi ,0)$ in terms of the phase ϕ = arg ζ(θ). Using the product of Pauli vectors (w⋅σ)(v⋅σ)=(w⋅v)1+i(w×v)⋅σ and the commutation relation $\left(\hat{n}\cdot \sigma )\sigma_{z}=-\sigma_{z}(\hat{n}\cdot \sigma \right)$ because ζ lies in the *xy* plane, the operator in the $I_{d}^{⋆}(z)$ integrand reduces to

$$\begin{array}{rl} e^{i(\zeta \cdot \sigma )z}\;i\partial_{\theta }\sigma_{z}\;e^{-i(\zeta \cdot \sigma )z} & =i\sigma_{z}[c1-is(\hat{n}\cdot \sigma )]\\ & \times [\frac{dc}{d\theta }1-i\frac{ds}{d\theta }(\hat{n}\cdot \sigma )-is(\frac{d\hat{n}}{d\theta }\cdot \sigma )] \end{array}$$ (25)

$$=i\sigma_{z}\{\frac{d}{d\theta }[\frac{1}{2}(c^{2}-s^{2})1-ics(\hat{n}\cdot \sigma )]-{is}^{2}(\hat{n}\times \frac{d\hat{n}}{d\theta })\cdot \sigma \}$$ (26)

where, for compactness, we abbreviated *c* = cos(∣ζ∣*z*) and *s* = sin(∣ζ∣*z*). When integrated against θ, the total derivative of a single-valued and periodic function vanishes by the fundamental theorem of calculus. For the remaining term, we note that $(\hat{n}\times \partial_{\theta }\hat{n})\cdot \sigma =(\partial_{\theta }\phi )\sigma_{z}$, leaving us with

$$I_{d}^{⋆}(z)=\frac{1}{2\pi }\int_{0}^{2\pi }\sin^{2}(|\zeta |z)\frac{d\phi }{d\theta }\;d\theta$$ (27)

In our experiment, we probe λ in the range of 600 to 750 nm. From simulation data, we know that over this range, the coupling constants *C*_1_ and *C*_2_ are well approximated by a linear dependence on λ, and the coupling ratio *C*_1_/*C*_2_ varies on the order of 1% per 100 nm. As a direct consequence, we can take, for every θ, the direction $\hat{n}$ as constant and the magnitude ∣ζ∣ as linear with respect to λ, with a slope between ∣∂_λ_*C*_1_ − ∂_λ_*C*_2_ ∣ = 31 m^−1^ and ∣∂_λ_*C*_1_ + ∂_λ_*C*_2_ ∣ = 92 m^−1^ per 100 nm. Hence, if we take measurements at a length *z* > π/31 m^−1^ ≈ 10 cm, then the quantity ∣ζ(λ) ∣ *z* samples point across the entire domain [0, π] of the sin^2^, over which sin^2^ has a mean value of 1/2. In our setup *z* = 17 cm, so we can average

$$\begin{array}{r} {\left\langle I_{d}^{⋆}(z)\right\rangle }_{\lambda }=\frac{1}{2\pi }\int_{0}^{2\pi }{\left\langle \sin^{2}(|\zeta |z)\right\rangle }_{\lambda }\;\frac{d\phi }{d\theta }\;d\theta =\frac{1}{2\pi }\int_{0}^{2\pi }\frac{1}{2}\frac{d\phi }{d\theta }\;d\theta =\frac{\nu }{2} \end{array}$$ (28)

From Eq. 25, we see that the average of our uncentralized weighted intensity difference is shifted from the winding number by a factor that depends explicitly on the labeling of the input core

$${\left\langle I_{d}(z)\right\rangle }_{\lambda }=\frac{\nu }{2}+m^{⋆}{\left\langle {\tilde{I}}_{d}(z)\right\rangle }_{\lambda }$$ (29)

When averaged over a very large range of wavelengths λ, this gauge freedom is fixed by the fact that ${\tilde{I}}_{d}(z)$ averages to zero

$${\left\langle {\tilde{I}}_{d}(z)\right\rangle }_{\lambda }=\frac{1}{2\pi }\int_{0}^{2\pi }(0\;1)\left(\;\sigma_{z}\;{\left\langle e^{-2i(\hat{n}\cdot \sigma )|\zeta |z}\right\rangle }_{\lambda }\right)\left(\begin{array}{c} 0\\ 1 \end{array}\right)\;d\theta =0$$ (30)

because *e*^2*ix*^ is centered at zero on the [0, π] domain. In our experiment, we pick a wavelength interval over which ${\left\langle {\tilde{I}}_{d}(z)\right\rangle }_{\lambda }=0$ so that 〈*I*_d_(*z*)〉_λ_ remains invariant under relabeling. In both approaches, we conclude that 2〈*I*_d_(*z*)〉_λ_ = ν, which is Eq. 3 in the "Results and discussion" section. However, the mean of the square of *I*_d_(*z*) does depend on the labeling *m*^⋆^. To estimate errors in our calculation of the winding number ν in an invariant way, we use the SD of the unweighted intensity difference ${\tilde{I}}_{d}(z)$, as plotted in Fig. 3B. This estimate for the error does not depend on the numbering of the unit cells. The error estimate measures the amplitude of the characteristic oscillations of the intensity difference as the wavelength is varied.
