# Supplementary material for: Topological supermodes in photonic crystal fiber
Source: Sci Adv. 2022 Dec 21;8(51):eadd3522. doi: 10.1126/sciadv.add3522 (PMC9770996; doi:10.1126/sciadv.add3522)
Supplement: Supplementary file 1 — Figs. S1 and S2 [file sciadv.add3522_sm.pdf]

Science Advances
AAAS

Supplementary Materials for

Topological supermodes in photonic crystal fiber

Nathan Roberts *et al.*

Corresponding author: Peter J. Mosley, p.mosley@bath.ac.uk; Anton Souslov, a.souslov@bath.ac.uk

Sci. Adv. **8**, eadd3522 (2022)
DOI: 10.1126/sciadv.add3522

The PDF file includes:

Figs. S1 and S2
Legends for movies S1 and S2

Other Supplementary Material for this manuscript includes the following:

Movies S1 and S2

Supplementary video captions

Supplementary Video 1: Wavelength dependence of topological edge mode. We vary the input wavelength from approximately 600nm to 500nm as we couple light into the central core and observe the output.

Supplementary Video 2: Wavelength dependence of topological bulk. We vary the input wavelength from approximately 600nm to 650nm as we couple light into core 6 and observe the output. Varying wavelength for input into a bulk core allows us to directly compute the topological invariant using Eq. (3).

Supplementary Figures

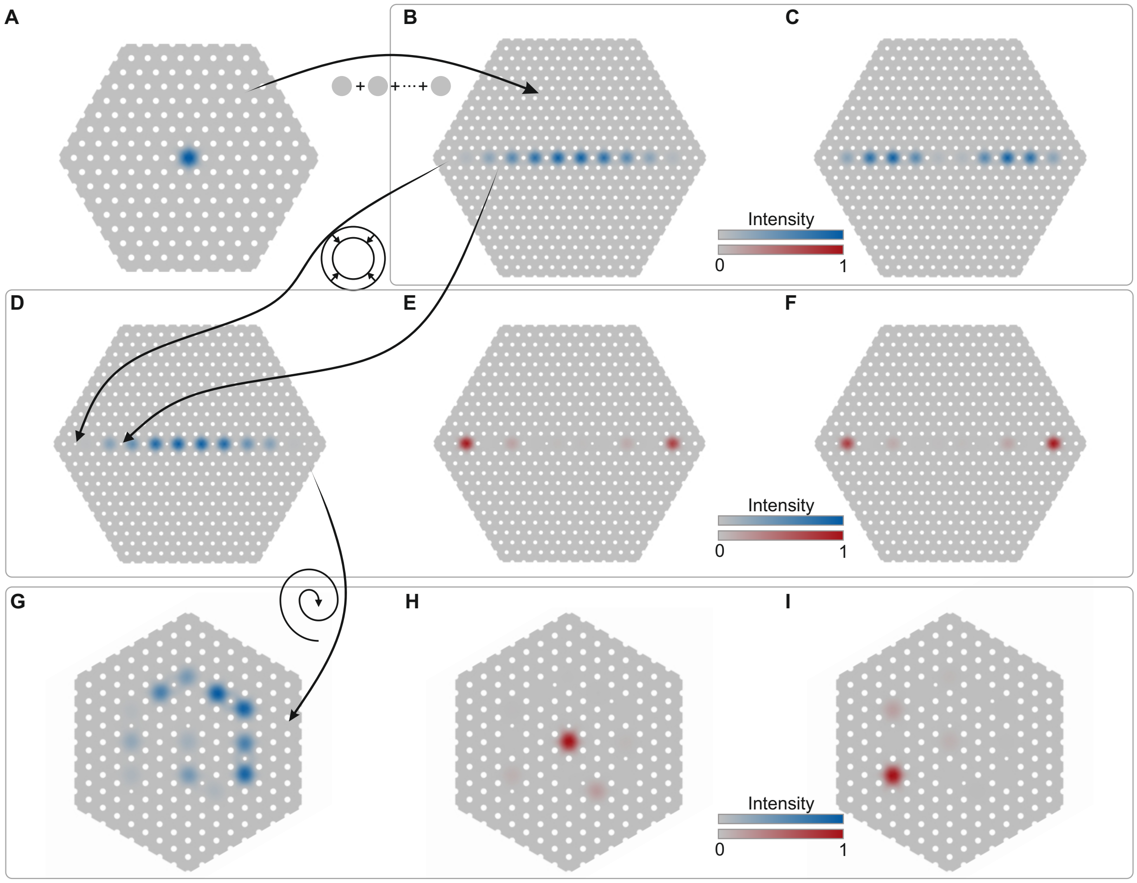

Figure S1: **Explanation of design process**. **A**, Simulation of an endlessly single mode PCF with a single light-guiding core. Placing multiple cores in the same cladding allows the fibre to support composite supermodes. All of these supermodes have profiles distributed across the entire chain (**B** and **C**). Shrinking the air holes between every other pair of cores creates a two-core unit cell with SSH-like coupling. **D**, Topological bulk mode supported by the multi-core chain with alternating air holes. **E**–**F**, Two degenerate topological edge modes supported by the fibre. The fibre cross-section in **D**–**F** can be reduced in size at fixed chain length by following the symmetries of the fibre. **G**, Topological bulk mode after twisting the chain into a spiral. **H**–**I**, Topological edge modes supported by the spiral chain.

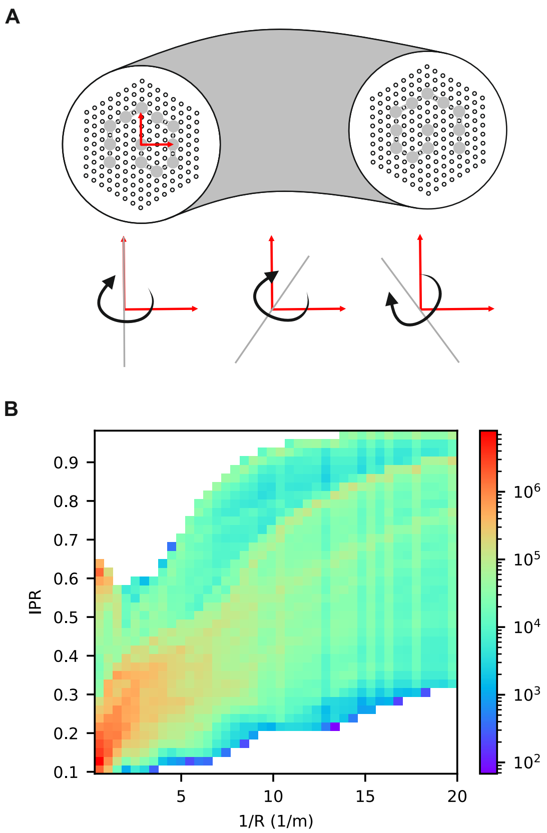

Figure S2: **Effects of bending around a random axis**. To determine the connection between on-site disorder and random bending, we first find the distance of every core from the random bend axis. **A**, Shows three examples of random bend axis (grey) relative to the fibre cross-section (red) in the lower left, middle, and right images. **B**, Random bending effects on IPR for 40,000 different bend angles at 200 different bend radii are plotted using a 2D histogram. The upper (lower) red spot towards the left side of the graph corresponds to the edge (bulk) modes. This shows that the modes can be separated into two distinct populations. Only edge modes were selected to be plotted in Fig. 3.
